# Salivary Trefoil Factor Family (TFF) Peptides and Their Roles in Oral and Esophageal Protection: Therapeutic Potential

**DOI:** 10.3390/ijms222212221

**Published:** 2021-11-12

**Authors:** Werner Hoffmann

**Affiliations:** Institute of Molecular Biology and Medicinal Chemistry, Otto-von-Guericke University Magdeburg, Leipziger Str. 44, 39120 Magdeburg, Germany; werner.hoffmann@med.ovgu.de

**Keywords:** saliva, artificial saliva, esophagus, TFF3, FCGBP, DMBT1, lectin, mucin, innate immune defense, xerostomia

## Abstract

Human saliva is a complex body fluid with more than 3000 different identified proteins. Besides rheological and lubricating properties, saliva supports wound healing and acts as an antimicrobial barrier. TFF peptides are secreted from the mucous acini of the major and minor salivary glands and are typical constituents of normal saliva; TFF3 being the predominant peptide compared with TFF1 and TFF2. Only TFF3 is easily detectable by Western blotting. It occurs in two forms, a disulfide-linked homodimer (Mr: 13k) and a high-molecular-mass heterodimer with IgG Fc binding protein (FCGBP). TFF peptides are secretory lectins known for their protective effects in mucous epithelia; the TFF3 dimer probably has wound-healing properties due to its weak motogenic effect. There are multiple indications that FCGBP and TFF3-FCGBP play a key role in the innate immune defense of mucous epithelia. In addition, homodimeric TFF3 interacts in vitro with the salivary agglutinin DMBT1^gp340^. Here, the protective roles of TFF peptides, FCGBP, and DMBT1^gp340^ in saliva are discussed. TFF peptides are also used to reduce radiotherapy- or chemotherapy-induced oral mucositis. Thus, TFF peptides, FCGBP, and DMBT1^gp340^ are promising candidates for better formulations of artificial saliva, particularly improving wound healing and antimicrobial effects even in the esophagus.

## 1. Introduction

### 1.1. Saliva

Saliva is a mixed body fluid produced in the oral cavity by different sources, i.e., three pairs of major salivary glands (parotid, sublingual, and submandibular glands) and several minor glands, such as labial and palatal glands. The parotid glands contain only serous acini, whereas all other glands are composed of a mixture of seromucous acini. Saliva is the fluid which has the first contact with ingested food and often the environment. It fulfills important functions for both nutrition (lubrication of the bolus, taste, and first steps of digestion) as well as for protection, particularly of the teeth, the oral epithelial barrier, but also the esophagus (reviews: [1,2,3,4]). For example, saliva has enzymatic, wound healing (cell migratory), and antimicrobial effects [1,4]. It is also essential for maintaining healthy oral microbiota [2,5].

Within the last two decades, the saliva proteome was subjected to a huge number of investigations, which often focused on highly specific aspects, such as certain diseases (also use of saliva as diagnostic fluid) and response to different stimuli, such as psychological stress (“stimulated saliva”) [6,7,8]. Recently, not only whole saliva was investigated, but also extra-vesicles-enriched saliva, which contains exosomes, i.e., extracellular vesicles, which probably originate from intracellular multivesicular bodies [9]. In normal saliva, more than 3000 proteins were identified with a relative abundance spanning about 14 orders of magnitude [6]. Only about 200 proteins represent about 90% in weight including proline-rich proteins, mucins, amylases, histatins, and statherins [5]. Of note, there are remarkable individual differences and the saliva composition is also subject to hormonal fluctuations and aging [3,4,5].

Typical salivary protein constituents are enzymes (such as amylases, lysozyme), protease inhibitors, growth factors such epidermal growth factor (EGF), antimicrobial peptides (such as histatins, defensins, cathelicidin), immunoglobulins (mainly secretory IgA and IgG), surfactant proteins, the agglutinin Deleted in Malignant Brain Tumor 1/gp340 (DMBT1^gp340^), IgG Fc binding protein (FCGBP), and secretory mucins (MUC5B, MUC7, MUC19) [2,5,10,11,12,13]. Many of these proteins also appear in tears [12]. Furthermore, there are proteins unique to this fluid, such as proline-rich proteins and statherins, which influence calcium phosphate chemistry and initial plaque formation; for example, statherin allows saliva to maintain a state of supersaturation concerning calcium phosphate [1,3,5]. Of note, most salivary proteins appear in protein families.

Mucins are glycoconjugates and the primary gel-forming components of mucus. Due to their special rheological properties, they are effective in lubricating the oral cavity including the teeth. They also play a key role for the innate immune defense of the oral cavity (review: [10]). The salivary mucins mainly appear in two molecular entities, the unusual low molecular mass mucin MUC7 (previously termed MG2) and the high-molecular-mass mucin MUC5B (previously MG1). Additionally, MUC19 has been identified on the transcript and protein level [10,14]. MUC5B, MUC7, and MUC19 are typically expressed in the mucous acini of the major and minor salivary glands [14]. MUC5B and MUC19 are typical gel-forming mucins, which evolved from a common ancestor with von Willebrand factor. In contrast, MUC7 lacks gel-forming properties. Both MUC5B as well as MUC7 form complexes with proline-rich proteins, statherins, and histatin 1 [10]. However, MUC5B and MUC7 differ remarkably in their binding characteristics with microbes [10]. MUC7 directly binds *Streptococcus* strains, but also to *Escherichia coli* and *Staphylococcus aureus*. Sialic acid residues from the carbohydrate moiety of MUC7 play a major role for binding of different microbes. In contrast, the binding of MUC5B to oral pathogens is limited and here protein–protein interactions seem to be important, e.g., for binding of *Haemophilus parainfluenzae*. MUC5B also reduces the virulence of *Candida albicans*. Furthermore, a mixture of MUC5B and MUC7 inhibits T cells from viral infections, i.e., HIV-1. Generally, there are two potential principles by which salivary mucins could protect the oral cavity; they could agglutinate microbes facilitating their removal or they disperse the microbes, hindering their transition into a virulent state [10].

Generally, saliva has a fundamental role for the innate immune system of the oral cavity, but also the esophagus and the delicate esophagogastric junction. Here, a wide range of different molecular mechanisms is used, such as adhesions (proline-rich proteins), agglutinins (e.g., mucins, DMBT1^gp340^), and antimicrobial peptides [1,2,4,5,10,11]. On the other hand, saliva is important for taste, digestion, and modulating the pH; it has pronounced rheological properties important for lubrication (mucins, etc.), but it also has to provide the necessary water by aquaporins (water channels) [1,2,4,5,14]. The importance of a functional salivary flow can be estimated from patients with a catastrophic loss of salivary function, such as patients with radiation therapy because of head and neck cancers, patients with congenital absence of salivary glands, and patients with Sjögren’s syndrome [3,4,9]. Thus, there is still a need for the development for better formulations of saliva substitutes, particularly with strongly improved antimicrobial properties (see Section 3.2).

### 1.2. Trefoil Factor Family (TFF) Peptides

More than two decades ago, expression of TFF peptides was also demonstrated in human salivary glands, where TFF3 transcripts were most abundant [15]. In saliva, only TFF3 is easily detectable by Western blotting, but not TFF1 or TFF2 [15,16,17]. The concentration of salivary TFF1 was reported to be about 20% of that of TFF3, whereas the salivary TFF2 concentration was below 1% of that of TFF3 [18]. In situ hybridization, laser microdissection, and immunohistochemistry localized TFF expression mainly in mucous acini of both the major and minor salivary glands, TFF3 expression being most abundant [14,19]. There were remarkable individual differences, also sometimes recognizing TFF3 in serous acini of submandibular glands [15]. TFF3 was also located in parotid gland ducts [20]. Furthermore, TFF3 (and also little TFF1 and TFF2) is synthesized in the oral mucosal epithelium [16,21]. Expression of TFF2 and TFF3 in oral mucosal tissue is downregulated in patients with oral squamous cell carcinoma and oral lichen planus [21,22]. Decreased salivary TFF3 levels were also observed in patients with obstructive sleep apnea and rhonchopathy [23]. Salivary TFF1 and TFF3 concentrations are reduced in patients with chronic periodontitis [24], whereas salivary TFF3 is elevated in children with oral mucositis [25]. TFF expression is increased in salivary gland tumors [26].

**Figure 1:**
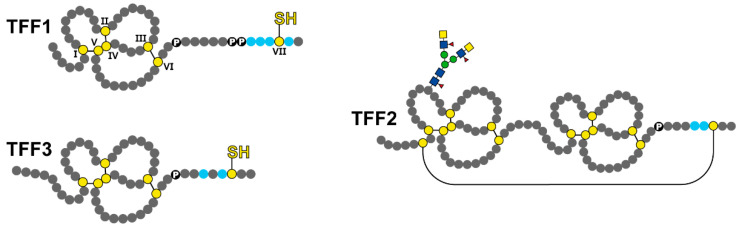
Schematic structures of the human TFF peptides TFF1, TFF2, and TFF3. Cysteine residues (C; numbering in Roman numerals) as well as the free thiol groups at Cys^VII^ in TFF1 and TFF3 are shown in yellow. TFF2 contains an N-linked carbohydrate moiety and an additional disulfide bridge between Cys-6 and Cys-104, which creates a circular structure. Additionally outlined are the proline residues (P) at the C-terminal outside the TFF domains. Acidic residues close to the C-terminal cysteine residues are shown in blue.

TFF peptides belong to a family of secretory lectins (i.e., sugar-binding proteins), which play different roles for mucosal protection (for recent reviews, see [27,28]). Thus, they are also considered as a protective shield of the oral cavity [29]. They consist of one (TFF1, TFF3) or two TFF domains (TFF2), each TFF domain being stabilized by three conserved disulfide bridges, i.e., Cys^I-V^, Cys^II-IV^, Cys^III-VI^ (Figure 1; for reviews, see [28,30,31]). Of note, and highly unusual for secretory peptides, TFF1 and TFF3 contain an odd number of cysteine residues, the seventh unpaired residue (Cys^VII^) being C-terminal and outside the TFF domain. The nucleophilicity of Cys^VII^ is modulated by neighboring acidic residues (change of pKa) as well as by steric exposure due to proline residues nearby (Figure 1). This is highly relevant for TFF1, which is directly flanked by four acidic residues and mainly occurs in the stomach as an unusual monomer.

TFF peptides are characteristically secreted by mucous epithelia and their glands. Here, exocrine secretion occurs, mainly together with different gel-forming mucins [27,28]. TFF1 is predominantly secreted from gastric surface mucous cells and TFF2 from gastric mucous neck and antral gland cells. In contrast, TFF3 is mainly synthesized in intestinal goblet cells, but also in most other mucous epithelia, such as the respiratory and urogenitary tracts and also the conjunctiva. Furthermore, TFF peptides also undergo endocrine secretion, where minute amounts are released from the central nervous system, the immune system, the endocrine pancreas, and the thyroid [27,28]. TFF peptides are linked to inflammation (review: [32]) and they play different roles in the mucosal innate immune defense (review: [33]). Here, I will discuss the role of salivary TFF peptides for the protection of the oral cavity, the esophagus, and also the delicate esophagogastric junction as well as their therapeutic potential, for example as constituents for improved formulations for artificial saliva. Major emphasis will be put on TFF3, as this is the predominant salivary TFF peptide in human.

## 2. Potential Roles of Salivary TFF Peptides

### 2.1. Potential Role of Salivary TFF1

Little TFF1 expression was detectable in mucous acini of submandibular, sublingual, and labial as well as in parotid glands [14,15,19]. Due to the minute amounts of TFF1 expected in the saliva, there are no protein data available thus far and it is not clear in which forms salivary TFF1 occurs. However, the situation in the human and murine stomach has been investigated in detail, where large amounts of TFF1 are synthesized [34,35]. Here, TFF1 mainly occurs as an unusual monomer, but also as a homodimer and as heterodimers with FCGBP and gastrokine 2 [34,35]. By analogy, one might expect that salivary TFF1 might also occur as a monomer, a homodimer, and a TFF1-FCGBP heterodimer, as FCGBP is a constituent of human saliva [12,17]. The hypothetical existence of a TFF1-FCGBP heterodimer would be also comparable with the TFF3-FCGBP heterodimer present in human saliva [17].

Monomeric TFF1 with its free and probably highly nucleophilic thiol group at Cys^VII^ (due to flanking acid residues and steric exposure; see Figure 1) could hypothetically act as a scavenger for reactive oxygen species (ROS) as discussed previously in detail [27,34,35,36]. In short, the free thiol at Cys^VII^ is masked by the four flanking acidic amino acids and thus escapes assembly (dimerization), retention, or degradation in the endoplasmic reticulum, similar to that described for Ig light chains [27]. This might be of biological significance as saliva is a rich source for ROS due to the generation of H_2_O_2_ by dual oxidase (DUOX) 2 from salivary glands and secreted lactoperoxidase, which produces microbicidal hypothiocyanite (OSCN^−^) anions [3,37]. Such a protective function as ROS scavenger might also be of special importance for the delicate esophagogastric junction, as reactive nitrogen species (RNS) are also formed there when nitrite from saliva meets the gastric juice [27]. As a prerequisite, salivary nitrate (NO_3_^−^), whose concentration is about 10–20 times higher than that in plasma due to the enterosalivary circulation, is reduced to nitrite (NO_2_^−^) by the oral microbiome [27,38,39]. After acidification in the gastric juice and disproportionation of the instable nitrous acid (HO-NO), the radical nitric oxide (NO) is formed, which is a gasotransmitter and can also react with O_2_^−^ to peroxynitrite (ONOO^−^) [27,38]. The latter is the prototype of a toxic RNS [27]. Thus, it could well be that salivary TFF1 might reduce the development of adenocarcinoma particularly at the delicate esophagogastric junction.

Additionally, monomeric TFF1 could be an intracellular chaperone involved in the correct folding of glycoproteins (such as mucins) in the endoplasmic reticulum [27,32]. In contrast, homodimeric TFF1 is able to interact as a lectin with *Helicobacter pylori* (for review, see [40]). Homodimeric TFF1 can also bind as a lectin to the gastric mucin MUC6 in vitro [34], which could also stabilize the inner gastric mucus layer particularly at the delicate esophagogastric junction.

The hypothetical formation of a salivary TFF1-FCGBP heterodimer is most interesting as it could play a role in the innate immune defense of the oral cavity and the esophagus comparable with TFF3-FCGBP (see Section 2.3). Furthermore, by analogy with the situation in the stomach, the formation of additional TFF1 heterodimers is possible [34,35].

### 2.2. Potential Role of Salivary TFF2

Only minute amounts of TFF2 are expressed in mucous acini of major and minor salivary glands [14,19] and there are no protein data on TFF2 in the saliva.

Based on studies from the stomach, where TFF2 is a major secretory peptide of mucous neck and antral gland cells together with the mucin MUC6, it is clear that TFF2 is a typical lectin specifically recognizing the GlcNAcα1→4Galβ1→R epitope at the non-reducing terminals of the MUC6 carbohydrate moiety (for review, see [41]). A prerequisite for the biosynthesis of this unusual sugar epitope is α1,4-*N*-acetylglucosaminyltransferase (A4GNT) and mice lacking this enzyme spontaneously develop antral adenocarcinomas [42]. Gastric TFF2 has probably a role in physically stabilizing the inner insoluble layer of the gastric mucus barrier (crosslinked mucous network) and thus can be considered as part of the gastric innate immune defense [27,28,33,43,44]. Furthermore, TFF2 has been reported to influence inflammatory processes probably via glycosylated basolateral receptors (for reviews, see [28,32]).

Currently, the biological role of salivary TFF2 is not established and it is not even clear if it is bound to mucins or if it exists in a non-bound form similar to that in the porcine pancreas [45]. There are no positive reports on MUC6 expression in salivary glands; MUC6 transcripts are absent in the esophagus, but they can easily be detected in the stomach starting at the Z-line [46]. There are also no reports on the expression of A4GNT in salivary glands and the esophagus. Thus, there is no indication for an interaction of salivary TFF2 with MUC6 or another mucin in the oral cavity or the esophagus. Possibly salivary TFF2 helps to protect the delicate esophagogastric junction. Furthermore, salivary TFF2 seems to bind as a lectin ligand to the carbohydrate moiety of various transmembrane receptors affecting, e.g., cell migration or an immune response (for reviews, see [28,32].

### 2.3. Potential Role of Salivary TFF3

**Figure 2:**
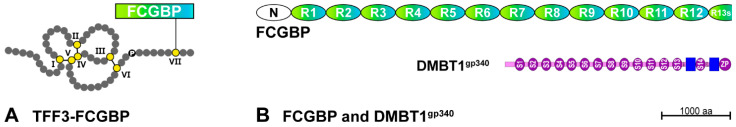
Schematic structures of the human TFF3-FCGBP heteromer (**A**) as well FCGBP and DMBT1^gp340^ (**B**). (**A**) TFF3 forms a three-leafed structure (TFF domain) by three disulfide bridges between Cys^I^ to Cys^VI^. The 7th cysteine residue is linked to the high molecular mass glycoprotein FCGBP (see panel **B**) via a disulfide bridge (not drawn to scale). Cysteine residues are shown in yellow. Additionally represented is a characteristic proline residue (P) separating the TFF domain from Cys^VII^. (**B**) Schematic structures of the high-molecular mass glycoproteins FCGBP and DMBT1gp^340^. Outlined are the various modules; some of them are repetitive and cysteine-rich, e.g., R1-R13s, S1-S14 (SRCR domains). Sale bar: 1000 amino acid residues.

In contrast to TFF1 and TFF2, TFF3 is easily detectable in human saliva and is mainly expressed in mucous acini of the major and minor salivary glands together with the mucin MUC5B [14,15]. About 20 to 80% of human salivary TFF3 exist in a high-molecular-mass form, which represents a TFF3-FCGBP heterodimer (Figure 2A) [17]. The low-molecular-mass fractions mainly represent different homodimeric TFF3 forms [17,47]. In the latter, a truncated TFF3 form was also characterized missing the C-terminal phenylalanine residue [17]. Degradation of salivary TFF3 might occur due to the presence of pepsin or bacterial proteases from the oral microbiome; of note, in 22% of healthy volunteers, pepsin/pepsinogen was detected in the saliva [48].

The biological function of homodimeric TFF3 in the saliva is not known currently. However, a protective role can be expected for both the oral epithelium as well as the esophagus [49]. Of note, the esophageal epithelium contains few submucosal glands, which also secrete TFF3 [46]. Taken together, the synthesis of TFF3 in salivary glands is reminiscent to TFF3 synthesis in glandular structures of the esophagus, the lung, and the cervix uteri, where TFF3 is co-secreted with the mucin MUC5B [46,50,51]. A possible protective role of TFF3 can be inferred from its weak motogenic and antiapoptotic activities (for review, see [27]). These cell migratory and survival effects are coordinately regulated in order to ensure synergy, e.g., wound healing (restitution) [52]. Recombinant human TFF3 dimer enhances migration of oral keratinocytes [53,54]. However, the motogenic effect is rather weak and might result from a lectin-triggered activation of basolateral transmembrane glycoproteins, such as CXCR4 and CXCR7 [55] (for reviews, see [27,28,56]). Of special note, the potential wound healing effect of TFF3 could be even enhanced in vivo by EGF (a typical constituent of human saliva) as synergistic motogenic effects were described with TFF peptides [57,58,59].

There are increasing indications that the high-molecular-mass TFF3-FCGBP heterodimer plays a key role for the mucosal innate immune defense (for review, see [33]). TFF3-FCGBP was originally characterized in the human intestine, where TFF3 is mainly expressed [60]. FCGBP is a secretory, repetitive, cysteine-rich glycoprotein comprising of about 5400 amino acid residues (see Figure 2B) [61], which is auto-catalytically processed at the 11 GD/PH sites with preferential processing at the six WGD/PH sites [60]. However, after processing, the proteolytic fragments are still linked by disulfide bridges [60]. The cysteine-rich repeats show similarity with von Willebrand factor and gel-forming mucins, such as MUC5B. FCGBP is ubiquitous in vertebrates [62] and is typically synthesized by numerous mucous epithelia and their glands, such as salivary glands, and thus is a constituent of many body fluids, such as saliva [12,14,61]. From fish to humans, FCGBP is a highly upregulated defense gene after bacterial or viral infections and it regulates pathogen attachment [63,64]. Thus, it is speculated that FCGBP is involved in the clearing of microorganisms and prevents bacterial infiltration [27]. It has even been suggested to act as a viral trap for HIV-antibody complexes [65]. Generally, FCGBP would be well suited to bind to salivary IgG, which mainly derives from blood through passive leakage [4,66]. Currently, the role of TFF3 in the TFF3-FCGBP heterodimer is not established, but TFF3 (and maybe also TFF1) could modulate the binding to microorganisms due to their lectin activities.

Of special note, TFF3 could also interact with another constituent of saliva, i.e., the glycoprotein DMBT1^gp340^, a known salivary scavenger and agglutinin (SALSA), or salivary agglutinin (SAG), which has important functions in innate immunity (for reviews, see [67,68]). DMBT1 appears in many body fluids and its glycosylation seems to be tissue specific [67,69]. Calcium-dependent binding of DMBT1^gp340^ and recombinant homodimeric TFF3 was observed in vitro [69]. In contrast, no binding was observed with monomeric TFF3 or TFF2 [69]. DMBT1^gp340^ is a repetitive glycoprotein containing mainly scavenger receptor cysteine-rich (SRCR) domains linked by short proline-rich segments (Figure 2B) [68]. On the one hand, it is able to aggregate *Streptococcus mutans* and *S. sanguis* as well as influenza A virus (maybe via its SRCR domains) promoting their clearance from the oral cavity [2,67]. On the other hand, DMBT1^gp340^ probably interacts via its mannose and fucose structures with the C-type lectin receptors DC-SIGN and Langerin, which prevented binding of *Candida albicans* and *Escheria coli* to these receptors [70]. Furthermore, DMBT1^gp340^ binds to a variety of host proteins, such as surfactant proteins, lactoferrin, MUC5B, galectin 3, and even TFF2 [67,71]. Currently, there are no reports demonstrating an interaction of TFF3 and DMBT1^gp340^ also in vivo. However, a lectin interaction might be possible and this would depend on the glycosylation status, which is tissue specific.

Furthermore, an interaction of homodimeric TFF3 (and even TFF3-FCGBP) with salivary mucins cannot be excluded at the moment and has to be considered thoroughly. This could be of importance as it could affect the viscoelastic properties of salivary mucus. Of special note, the TFF3 concentration in the cervical mucus plug was reported to be correlated with the viscoelastic properties [72]. Remarkably, the gel-forming mucin MUC5B is a major constituent in both the cervicovaginal mucus barrier as well as the saliva [10,73]. Generally, a lectin interaction is most likely as homodimeric TFF3 has documented lectin activities (for reviews, see [27,28]).

Taken together, all three TFF3, FCGBP and DMBT1^gp340^ are synthesized by mucous epithelia and are involved in mucosal innate immune defense mechanisms. Generally, they could form a complex interaction network. Of special note, *Dmbt1*-deficient (*Dmbt1*^KO^) mice show the same phenotype as *Tff3*^KO^ mice, i.e., they react extremely sensitively in a dextran sulfate sodium (DSS)-induced colitis model [67,74]. Here, particularly TFF3, which together with FCGBP is mainly secreted by intestinal goblet cells, seems to strengthen the outer colonic mucus barrier by inhibiting microbial attachment, supporting their clearance, and inhibiting penetration of the inner mucus layer (for reviews, see [28,33]). A similar mechanism could well protect the oral cavity and the esophagus.

### 2.4. Summary

Taken together, the protective roles of TFF peptides seem to differ at the mechanistic level [27]. TFF3 is the major salivary TFF peptide and large amounts exist as a TFF3-FCGBP heterodimer [17]. Thus, TFF3 and FCGBP are expected to play a key role in oral and esophageal protection. In Table 1, the possible roles of salivary TFF peptides are summarized.

## 3. Therapeutic Potential and Clinical Perspectives

### 3.1. Saliva, Esophagus and Esophagogastric Junction

Saliva contains high amounts of nitrate, about 10–20 times higher than in plasma [38]. This leads to the formation of nitric oxide and peroxynitrite, particularly at the delicate esophagogastric junction as this is the primary site of acidification by the gastric juice (see also Section 2.1) [38,39]. This is probably an important factor for the development of adenocarcinoma at the esophagogastric junction [39]. Of note, in patients with gastro-esophageal reflux disease, the anatomical location where saliva meets the gastric juice is somewhat changed towards the distal esophagus [39]. Remarkably, TFF3 expression is increased at the esophagogastric junction in gastro-esophageal reflux disease [75]. Furthermore, saliva also seems to be a pivotal player in the pathogenesis of oropharyngeal cancer [4,76]. Here, ROS play a key role and the addition of glutathione with its free thiol group as antioxidant is protective against damages by aldehydes from cigarette smoke [76]. Additionally, monomeric TFF1 may also be protective here (see Section 2.1).

In humans, TFF3 is the predominant TFF peptide in the salivary glands, and saliva and relatively little TFF3 (and no TFF1 and TFF2) is synthesized in the few esophageal submucosal glands [46]. Thus, the human esophagus seems to rely on protection by the saliva. Of special note, and in contrast, the esophagus of the frog *Xenopus laevis* is protected by massive own synthesis of an ortholog of TFF2, i.e., xP4, by esophageal goblet cells [44,77].

### 3.2. TFF Peptides and Their Use in Chemo- and Radiotherapy and in Artificial Saliva

In the past, TFF peptides have been repeatedly used to protect mucous epithelia from damage (for compilation and reviews, see [78,79,80]). For example, *Tff3*^KO^ mice were more susceptible to chemotherapy- or radiotherapy-induced intestinal damages and oral application of recombinant TFF3 reduced intestinal mucositis [81]. Subsequently, an oral spray of human dimeric recombinant TFF3 was successfully used in a phase II study to treat colorectal cancer patients in order to reduce chemotherapy-induced oral mucositis [82]. In another attempt, all three TFF peptides delivered by genetically modified *Lactococcus lactis* were shown to prevent DSS-induced colitis in mice [83]. Later, a mouth rinse formulation of *L. lactis*-secreting TFF1, coded AG013, was applied to reduce radiation-induced oral mucositis in a hamster model [84]. Finally, these positive results were confirmed in a phase Ib study, where patients with locally advanced head and neck cancer (LAHNC) were treated with AG013 during chemotherapy (35% reduction in percentage of days with ulcerative oral mucositis) [85]. Taken together, the protective effects from chemotherapy- or radiotherapy-induced oral mucositis might be due to the weak motogenic and antiapoptotic effects of TFF peptides as well as by anti-inflammatory effects, which could be triggered by binding to the carbohydrate moiety of numerous transmembrane glycoproteins (for reviews, see [27,28,32]).

Pharmacological inhibition of TFF3 dimerization by a synthetic drug was reported to enhance the sensitivity of colorectal carcinoma to chemotherapy [86]. Thus, on the one hand, TFF peptides (especially homodimeric TFF3) can be expected to protect from chemotherapy- or radiotherapy-induced oral mucositis. On the other hand, the inhibition of TFF3 dimerization may positively support the effect of chemotherapy. Thus, the application particularly of homodimeric TFF3 in oral cancer patients during chemotherapy needs caution and should be investigated in detail as it has the potential to act as a double-edged sword.

TFF3, together with FCGBP, could play a major role in the innate immune defense of the oral cavity and the esophagus (see Section 2.3 and Table 1). Thus, the application of TFF3-FCGBP and/or FCGPB seems to be a novel promising strategy to protect the oral cavity from microbial infections, which are typical side effects of radiotherapy and chemotherapy [87]. In addition to radiotherapy or chemotherapy, reduced saliva production may also be caused by certain diseases, medications, or aging, which leads to a chronic sensation of a dry mouth called xerostomia [3,88]. Currently, there is still a need for the development of better formulations of saliva substitutes, i.e., artificial saliva, and more sophisticated strategies are needed [88]. Besides rheological and lubricating effects, artificial saliva should support wound healing and have antimicrobial properties [88]. Particularly the latter two properties are important for the maintenance of a healthy oral epithelial barrier [89]. A combination of TFF peptides, FCGBP, and DMBT1^gp340^ would be promising to support both wound healing and antimicrobial defense. Currently, there is a formulation commercially available from a porcine gastric mucin preparation, which contains relatively large amounts of TFF2, but no detectable levels of TFF1 or TFF3 [90]. However, TFF2 is the least abundant TFF peptide in human saliva and thus this formulation does not reflect the natural situation of TFF peptides in human saliva.

## Figures and Tables

**Table 1:** Possible roles of salivary TFF peptides in oral and esophageal protection. The different forms of TFF3 are shown off bold as TFF3 is the predominant TFF peptide in the saliva.

TFF Peptides	Possible Role of Salivary TFF Peptides	References
TFF1 monomer	Scavenger for ROS and RNS	[27,34,35,36]
	Intracellular chaperone (ER)	[27,32]
TFF1 homodimer	Interaction with *H. pylori*	[40]
	Binding to MUC6 (esophagogastric junction)	[34]
TFF2	Weak motogenic activity	[27,28,32,33]
	Anti-inflammatory effect	[28,32]
	Binding to MUC6 (esophagogastric junction)	[27,33,41]
**TFF3 homodimer**	Weak motogenic activity	[27,28,32,33,53,54]
	Binding to DMBT1^gp340^ (at least in vitro)	[33,69]
**TFF3-FCGBP**	Regulation of pathogen attachment and clearing of microorganisms	[27,33]

## Abbreviations

DMBT1	Deleted in Malignant Brain Tumor 1
DSS	Dextran sulfate sodium
FCGBP	IgG Fc binding protein
ROS	Reactive oxygen species
SRCR	Scavenger receptor cysteine-rich
TFF	Trefoil factor family
